# Bone defect reconstruction with a novel biomaterial containing calcium phosphate and aluminum oxide reinforcement

**DOI:** 10.1186/s13018-020-01801-8

**Published:** 2020-07-29

**Authors:** Alexander M. Keppler, Maximilian M. Saller, Alberton Paolo, Westphal Ines, Heidenau Frank, Schönitzer Veronika, Böcker Wolfgang, Kammerlander Christian, Schieker Matthias, Aszodi Attila, Neuerburg Carl

**Affiliations:** 1 Department of General, Trauma and Reconstructive Surgery, University Hospital of the Ludwig-Maximilians-University Munich, Campus Großhadern, Marchioninistraße 15, 81377 Munich, Germany; 2 Novartis Institute for Biomedical Research, Basel, Switzerland; 3 BioCer GmbH, Bayreuth, Germany; 4 LivImplant GmbH, Starnberg, Germany

## Abstract

**Background:**

Reconstruction of metaphyseal fractures represents a clinical challenge for orthopedic surgeons. Especially in osteoporotic bone, these fractures are frequently accompanied by osseous substance defects. In order to ensure rapid mobilization of patients, high stability requirements must be met by osteosynthesis. Various bone graft materials have been introduced in the past, such as autologous bone or exogenous bone substitute materials. These are used as bone void fillers or as augmentation techniques to ensure safe fixation of osteosynthesis. New calcium phosphate-based bone void-filling materials could be a promising alternative to autologous bone or to the currently and widely used polymethylmethacrylate (PMMA)-based cement. The aim of this study was to evaluate a novel paste-like bone void filler in vivo and in vitro with regard to biocompatibility and osteoconductivity.

**Methods:**

In addition to in vitro testing of cell compatibility using pre-osteoblasts (MC3T3-E1), 35 Wistar rats were treated in vivo with implantation of various material mixtures based on calcium phosphate and aluminum oxide reinforcement in a metaphyseal drill hole defect. After 4 weeks, an examination by micro-computed tomography (μCT) and histology was performed.

**Results:**

The in vitro analysis showed good biocompatibility with a high cell survival of osteoblasts. In the in vivo experiments, a significantly higher bone ingrowth compared to the empty defect was shown by μCT and histological analysis. Here, the group receiving material reinforced with aluminum oxide (Al_2_O_3_) showed a bone volume/tissue volume (BV/TV) of 89.19% compared to a BV/TV of 83.14% for the empty defect (*p* = 0.0013). In the group treated with a polysaccharide matrix, no increase in BV/TV was observed given a mean ratio of 80.14%. Scoring of histological sections did not reveal a significant difference between CaP and CaP that was substituted with Al_2_O_3_.

**Conclusion:**

The results of this study show an encouraging first step towards the development of new pasty, bone void-filling materials. We demonstrated that a new paste-like bone-filling material, based on calcium phosphate granulates and aluminum oxide to provide strength, exhibits good biocompatibility and osteoconductivity. Further biomechanical test in an osteoporotic animal model will have to be performed, to prove feasibility in metaphyseal defects.

## Introduction

Fracture fixation of the weight-bearing skeleton can be challenging. Early loading and precise fracture reduction have a major impact on the long- and short-term outcome, especially in older patients. Particularly, metaphyseal fractures with bone defects in osteoporotic bone remain a continuous surgical problem. Along with the presence of osteoporosis, the incidence of metaphyseal and vertebral osteoporotic fractures is constantly rising [1]. Almost every second women and 20% of men experience an osteoporotic fracture in their life [2, 3]. Due to the characteristics of osteoporotic bones, including reduced trabecular thickness and limited implant fixation, the clinical management of osteoporotic fractures is an important issue in orthogeriatric traumatology. Given the reduced bone quality and prolonged fracture healing, augmentation techniques may be necessary to ensure fracture reduction and fast mobilization.

Various techniques for the reconstruction of bone defects and augmentation of osteosynthesis have been described so far. Bone cements based on polymethylmethacrylate (PMMA) have been used very successfully for augmentation of vertebrae and other osteoporotic fractures for more than half a century. PMMA is used to improve anchorage of osteosyntheses or as a bone void filler. Although the stability and tissue compatibility of PMMA cement are excellent, it is not biodegradable, and during the exothermic polymerization process, the cement can heat up to 80 °C. This temperature is higher than the critical level of protein denaturation in the body and could lead to osteonecrosis [4–6]. Additionally, foreign body reactions and the inhibition of endogenous bone formation have been described for PMMA-based cement materials [7–9].

To avoid problems that are associated with bone substitute materials, it is necessary to develop bioresorbable and osteoconductive biomaterials, which can be used for augmentation and for reconstruction of bone defects. Their main purpose is to increase bone/implant interface and to fill voids in metaphyseal bone, as autologous bone grafts are accompanied by some disadvantages [10]. Thus, autologous bone grafts can be associated with a prolonged operation time and greater surgical trauma, which can be disadvantageous especially in older patients with limited bone regenerative capacity [11].

Therefore, requirements for an ideal augmentation and bone void-filling material depend on the clinical requirements, such as easy handling and low costs, but also on biological parameters. Ideally, bone reconstructive materials imitate natural bone, enhance osteoinduction, and provide a high primary stability. Biomaterials based on calcium phosphate (CaP) and hydroxyapatite (HA) are promising candidates for new bone void-filling materials [12]. Such sintered materials offer excellent biological properties and the required strength to stabilize and support load-bearing on the defect. Different mixtures of calcium phosphates have already been produced and successfully tested in vitro and in vivo [13].

In this study, we utilized a rat model of metaphyseal bone defect to gain general information about the features of a novel biomaterial containing CaP and aluminum oxide (Al_2_O_3_) particles to increase strength [14]. In the field of arthroplasty, aluminum oxide has been used successfully for a long time [15]. These granules are embedded in a paste-like matrix of polysaccharides, which ensures easy transport of these particles during application and their homogeneous distribution in the defect site. The goal of this study was to assess biocompatibility and osteoconductivity of a novel paste-like bone void-filling material under in vitro and in vivo conditions (rat model) with subsequent μCT analysis and histology. The aim of this mixture is to combine the osteoconductive properties of CaP granules with the mechanical strength of Al_2_O_3_ granules. The highly stable aluminum oxide could, for example, bring advantages for the application in vertebroplasty and provide a high primary pressure stability. The goal of this study was to assess biocompatibility and osteoconductivity of a novel paste-like bone void-filling material under in vitro and in vivo conditions (rat model) with subsequent μCT analysis and histology.

## Material and methods

### Animal experiments

Animal experiments were approved by the local government committee (Regierung von Oberbayern, file reference: 55.2-1-54-2531-112-10). In brief, 35 mature female rats (Wistar, 10 weeks old, 200–220 g body weight (BW), Charles River Laboratories, Sulzfeld, Germany) were anesthetized by intramuscular injection of fentanyl (0.005 mg/kg body weight (BW)), midazolam (0.2 mg/kg BW), and medetomidin (0.015 mg/kg BW). Rats were housed in groups of two per cage in a temperature- and air-controlled environment, with 12 h light/dark cycle, on chipped wood litter bedding and fed ad libitum. In the present pilot study, an established small animal model was chosen in order to facilitate comparison with findings from other groups. Therefore, grown female Wistar rats were used at the age of 10 weeks, as these animals are frequently used to assess biocompatibility under in vivo circumstances and hormonal effects are reduced at that age [16].

### Surgery

Surgery was performed under aseptic conditions and with standard surgery equipment. During the procedure, all animals were placed on a heating plate to prevent hypothermia and provided with 100% oxygen [17]. Rat legs were shaved, and the disinfected skin covered with a surgical drape. A lateral standard approach to the right distal femur was made with a #11 size blade to expose the distal shaft and the femoral condyles. The muscle and the patellar tendon were protected with retractors. A monocortical drill hole was created directly above the lateral condyle using a dental bur (diameter of 2.0 mm) fixed to a dental drilling unit (W&H Dentalwerk, Buermoos, Austria). The dental drill was cooled with physiological saline solution during drilling. We paid attention to protect the joint capsule of the knee. The drill hole volume of approximately 6.2 mm^3^ was flushed with physiological saline solution and filled up with one of the combinations of the bone-filling materials. Animals without bone-filling material served as controls. For application of the materials, a tuberculin syringe (Omnifix®, B.Braun, Melsungen, Germany) without needle was used.

The musculature was sutured using resorbable suture material (Vicryl 5-0, Ethicon GmbH, Norderstedt, Germany), and the skin was closed with non-resorbable suture material (Prolene 5-0, Ethicon GmbH, Norderstedt, Germany). Subsequently, anesthesia was antagonized by a subcutaneously administrated mixture of flumazenil (0.2 mg/kg BW), naloxon (0.12 mg/kg BW), and atipamezol (0.75 mg/kg BW). For pain relief, rats obtained buprenophin (0.05 mg/kg BW) subcutaneously for the following 4 days every 8 h for the first three postoperative days. All rats showed perfect mobility 4 h postoperatively.

After 4 weeks, animals were sacrificed with CO_2_ asphyxiation_._ The operated femur was explanted, fixed in 4% paraformaldehyde (PFA) in PBS for 24 h, and stored in PBS until μCT scans and histology.

### Composite materials

Different compositions were used as bone void-filling materials (Table 1).

**Table 1:** Composition of bone substitute materials used in this study

Bone substitute material	PM [wt%]	H_2_O [wt%]	CaP [wt%]	Al_2_O_3_ [wt%]
PM	12.30	87.70	–	–
CaP	–	–	100.00	–
PM/CaP	5.59	39.86	54.55	–
PM/CaP and Al_2_O_3_	5.59	39.86	13.64	40.91

*PM* polysaccharide matrix (paste-like base), *CaP* biphasic calcium phosphate (granulate), *Al*_*2*_*O*_*3*_ aluminum oxide. CaP was used as dry granulates

**Fig. 1:**
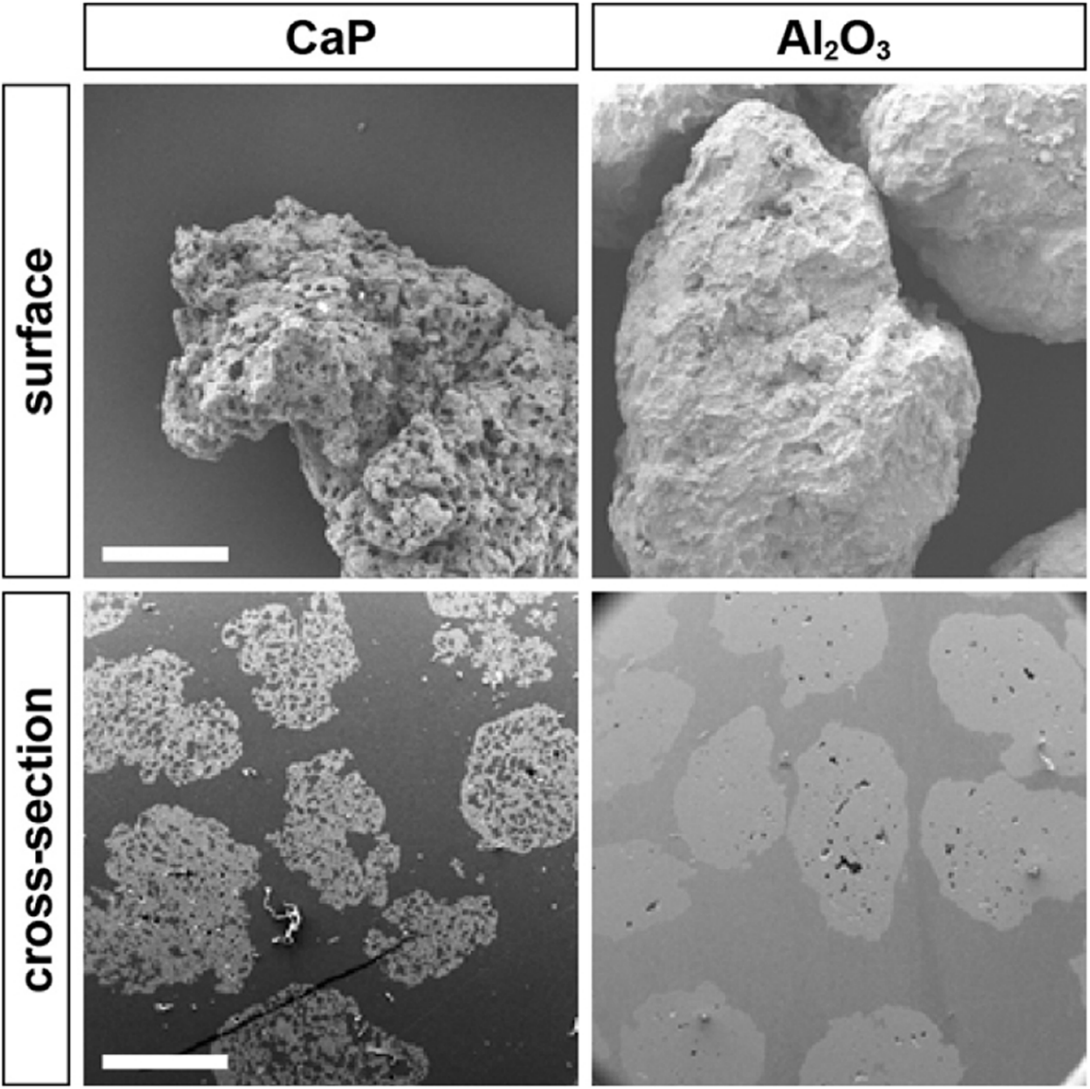
Structure of the filling material. Scanning electron microscopy. Scale bars—surface 500 μm, cross-section 1000 μm

Granulates with a size range of 200 to 800 μm were provided by BioCer (BioCer Entwicklungs GmbH, Bayreuth, Germany) and used to fill the defects. CaP consists of a porous hydroxyapatite/tricalcium phosphate (HA/TCP) granulate made up of 60% HA and 40% TCP (Fig. 1). Furthermore, to achieve increased strength, a mixture with a proportion of aluminum oxide powder (Al_2_O_3_, Fig. 1) was also tested. In order to transport the granules into the bone defect, a polysaccharide-based matrix (PM) based on potato carboxymethyl starch was applied as a carrier. This matrix also ensures the homogeneous distribution of the materials at the defect site.

### Scanning electron microscopy

To visualize material characteristics of the utilized materials, scanning electron microscopy images (Fig. 1) of the surface and cross-sections were created using a FEI Quanta 200 microscope. Therefore, granulates were mounted with carbon adhesive on sample plates and sputtered with gold (45 s, Sputter Coater 108 auto, Cressington). Samples were either directly imaged (surface images) or prior to imaging embedded in epoxy resin (EpoFix Kit, Struers), grinded and polished on a Struers DAP-7. While surface images were acquired at 20 kV and a × 100 magnification, cross-section images were acquired at 15 kV and a × 50 magnification.

### μCT

Morphometric analysis of the treated metaphyseal defects was carried out via μCT with a volume of interest (VOI) focusing the distal femora, similar to the VOI chosen for semi-quantitative histological analysis (Fig. 5).

Formation of bone within the defect was quantified using a SkyScan 1072 (Bruker, Arteselaar, Belgium) μCT analysis. Resolution was set at 8.68 μm operating at a source voltage of 49 kV and 200 mA. In order to illustrate bone ingrowth, a circular volume of interest was placed at the center of the drill hole with a diameter of 2.0 mm. Thus, a constant fixed threshold level (minimum gray value of 5 and maximum gray value of 200; threshold 99–245 units, 8 bit image) was used for rendering 3D images and to investigate the microstructure within the drill hole. Epiphyseal trabecular bone volume per tissue volume (BV/TV), trabecular thickness (Tb.Th), and trabecular number (Tb.N) were calculated using Skyscan Software (Bruker, Arteselaar, Belgium).

### Histological evaluation

Fixed femora including the bone-filling materials were used for undecalcified bone histology. After μCT scanning, bone samples were dehydrated in a graded alcohol series. The femora were embedded in Technovit-9100 New (Heraeus-Kulzer, Wehrheim, Germany). After the hardening process, 100-μm discs were cut with a diamond saw and then manually ground down to a thickness of 20 μm. Thereafter, 20-μm slices were stained with Masson-Goldner’s trichrome to investigate new bone formation. Sections were imaged using an inverted optical microscope (AxioObserver Z1, Zeiss, Oberkochen, Germany). A semi-quantitative assessment for bone ingrowth published by Hamilton et al. was performed by three blinded observers [17]. The semi-quantitative histological scoring consists of three components. Bone cross-sectional area, bone maturity, and cell activity were evaluated on a 0–4-point scale. Furthermore, attention was paid to the presence of inflammatory cells.

### Biocompatibility tests

Using pre-osteoblasts (MC3T3-E1), which were applied to various materials, biocompatibility was assessed. For the assessment of viability, 40,000 cells in complete culture medium (89% DMEM high glucose, 10% FBS, 1% penicillin/streptomycin, all from Life Technologies, Carlsbad, USA) were seeded onto 100 mg bone substitute material, and after 24 h, a live-dead assay was performed. Briefly, cell-seeded scaffolds were incubated with 10 μM carboxyfluorescein diacetate succinimidyl ester (in phosphate-buffered saline—PBS) for 5 min, rinsed two times in PBS, and dead cells were subsequently briefly counterstained with 0.5 mg/ml propidium iodide (both from SigmaAldrich, St. Louis, USA). Samples were rinsed three times in PBS and imaged with an inverted epifluorescence microscope (AxioObserver Z1, Zeiss, Oberkochen, Germany). To quantify the metabolic activity, we performed a WST-1 assay (Roche, Basel, Switzerland) by incubating the cell-seeded material as previously described [16]. Cells that were seeded on polymerized PMMA served as a control.

### Statistical evaluation

For all in vitro experiments, at least three independent runs were carried out in triplicates. Statistical analysis was performed using GraphPad Prism 5 (GraphPad Software, La Jolla, USA). Normal distribution was determined by a Shapiro-Wilk test, and significances were calculated using a one-way ANOVA test. Post hoc analysis was performed with a Tukey test. If the data were not normally distributed, the Kruskal-Wallis test was used. A value of *p* < 0.05 was considered significant. While in vitro results were expressed as mean with standard deviation (SD), in vivo data are shown as box plots (box: median, 25%, and 75% quartiles; error bars: minimum, maximum).

## Results

A total of 35 animals were included for evaluation of the results divided into different groups as listed below. Due to a postoperative wound healing disorder, two animals had to be sacrificed. Table 2 shows the group distribution after 4 weeks.

**Table 2:** Group composition after 4 weeks

Group	Number of animals
Empty defect	7
PM	6
CaP	6
PM/CaP	7
PM/CaP and Al_2_O_3_	7

### Murine osteoblast precursor cells show an unaffected viability and metabolic activity on aluminum oxide granulates

**Fig. 2:**
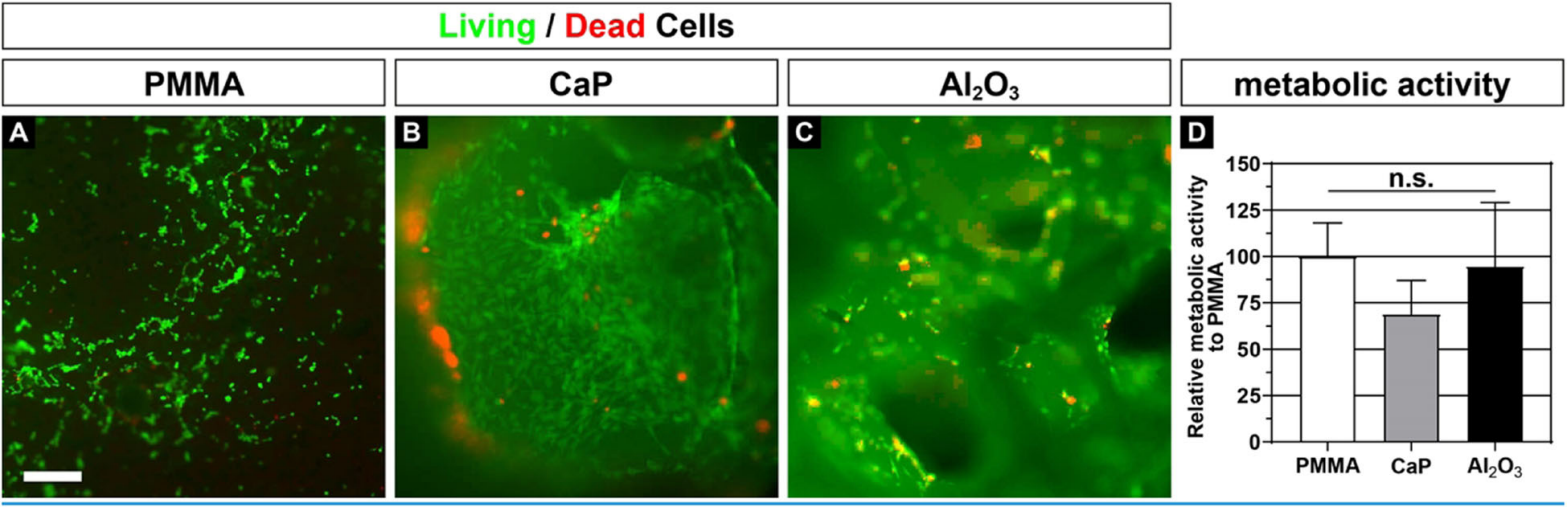
**a**–**c** MC3T3-E1 cells exhibit good survival rate on PMMA control, CaP, and Al_2_O_3_. Alive cells fluoresce in green, whereas dead cells fluoresce in orange. **d** Metabolic rate measured by WST-1 assay shows no significant difference in metabolic activity. n.s., not significant

Live/dead assay on MC3T3-E1 cells (Fig. 2) showed good cell survival on the two main components CaP and Al_2_O_3_. However, the comparison of cell metabolism by a WST-1 assay revealed a slight, but not significant, lower metabolic activity on pure CaP, when compared to PMMA. In contrast, cells that were seeded on Al_2_O_3_ demonstrated nearly the same metabolic activity in comparison to cells on PMMA.

### Histological evaluation

**Fig. 3:**
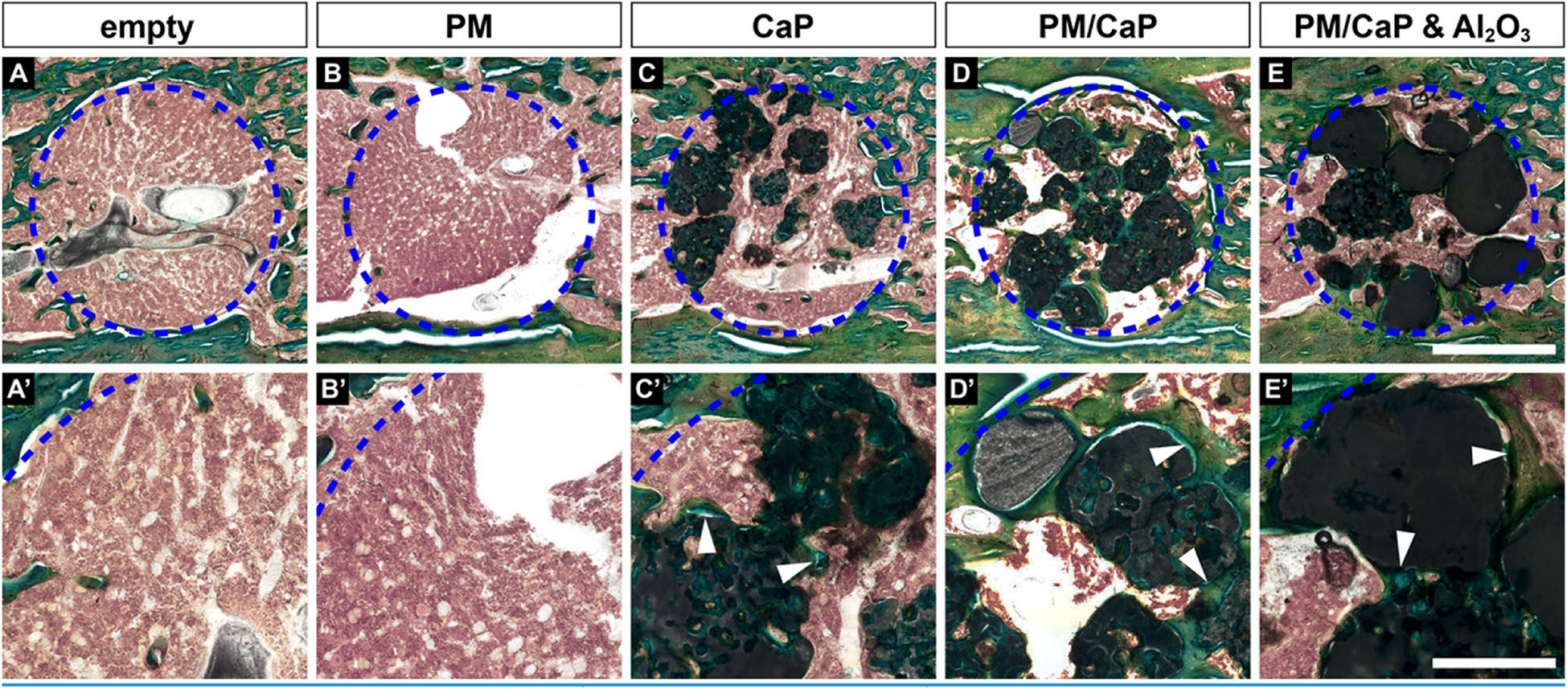
Representative histological sections (Masson*-*Goldner trichrome) demonstrate a plain defect in empty and PM-treated defects (**A**, **A’** and **B**, **B’**). In contrast, ingrowth of the surrounding bone (arrowheads) into all CaP-filled holes could be observed (**C**–**E** and **C’**–**E’**). Scale bar: **A**–**E** 1 mm, **A’**–**E’** 300 μm

The Masson-Goldner trichrome staining on sections of rat femora (Fig. 3a–e, A’–E’) indicates that implantation of all material composite cements was well tolerated in the defect sites after 4 weeks. Necrosis was not detected. Histologically, newly formed trabeculae could be detected in animals with bone substitutes (Fig. 3c’–e’, arrowheads). Furthermore, no ectopic bone formation or inflammatory cells were found.

**Fig. 4:**
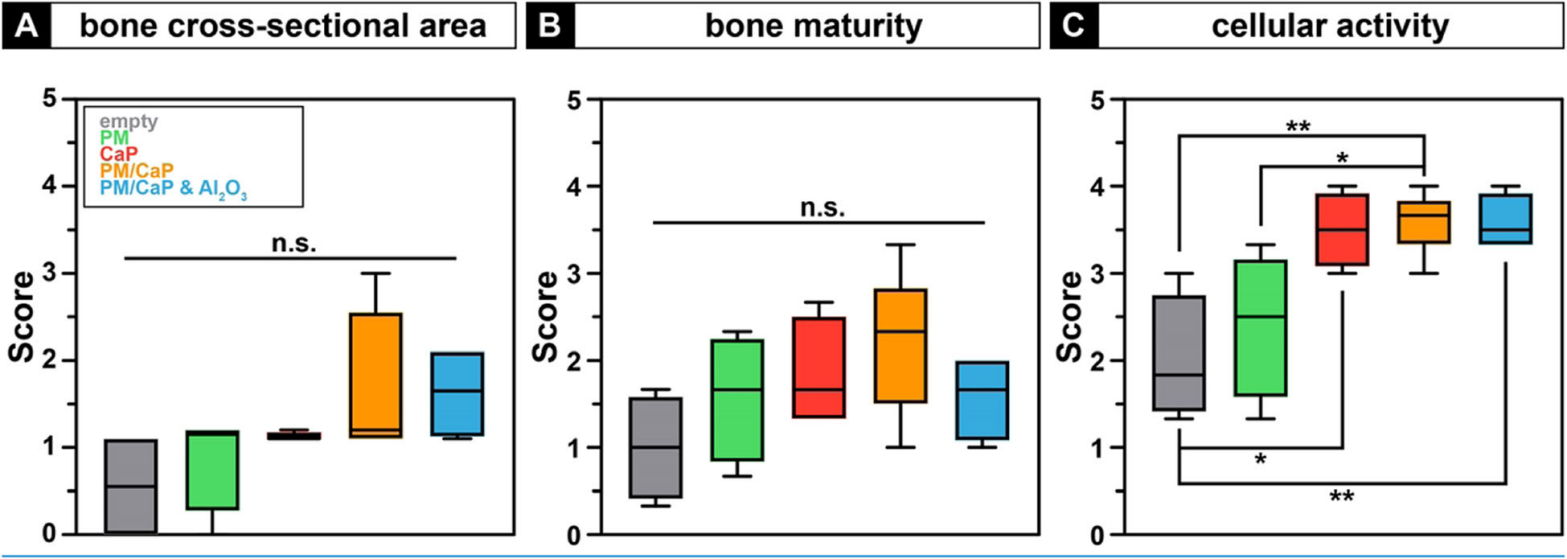
Result of the histological scoring. For the bone cross-sectional area, there is no significant difference (**a**). Also, for bone maturity, no significant difference can be observed between the groups (**b**). The osteogenic cellular activity is significantly higher in all granulate-treated defects compared to the empty defect and the control group (**c**). *p ≤ 0.05; **p ≤ 0.01; n.s., not significant

Histological scoring revealed a significant increase of cellular activity in the groups CaP, PM/CaP, or PM/CaP and AL_2_O_3_, when compared to animals with an empty or PM-filled defect (Fig. *4*c). However, there was no intergroup difference for the bone cross-sectional area (Fig. *4*a) or bone maturity (Fig. *4*b).

### μCT analysis

**Fig. 5:**
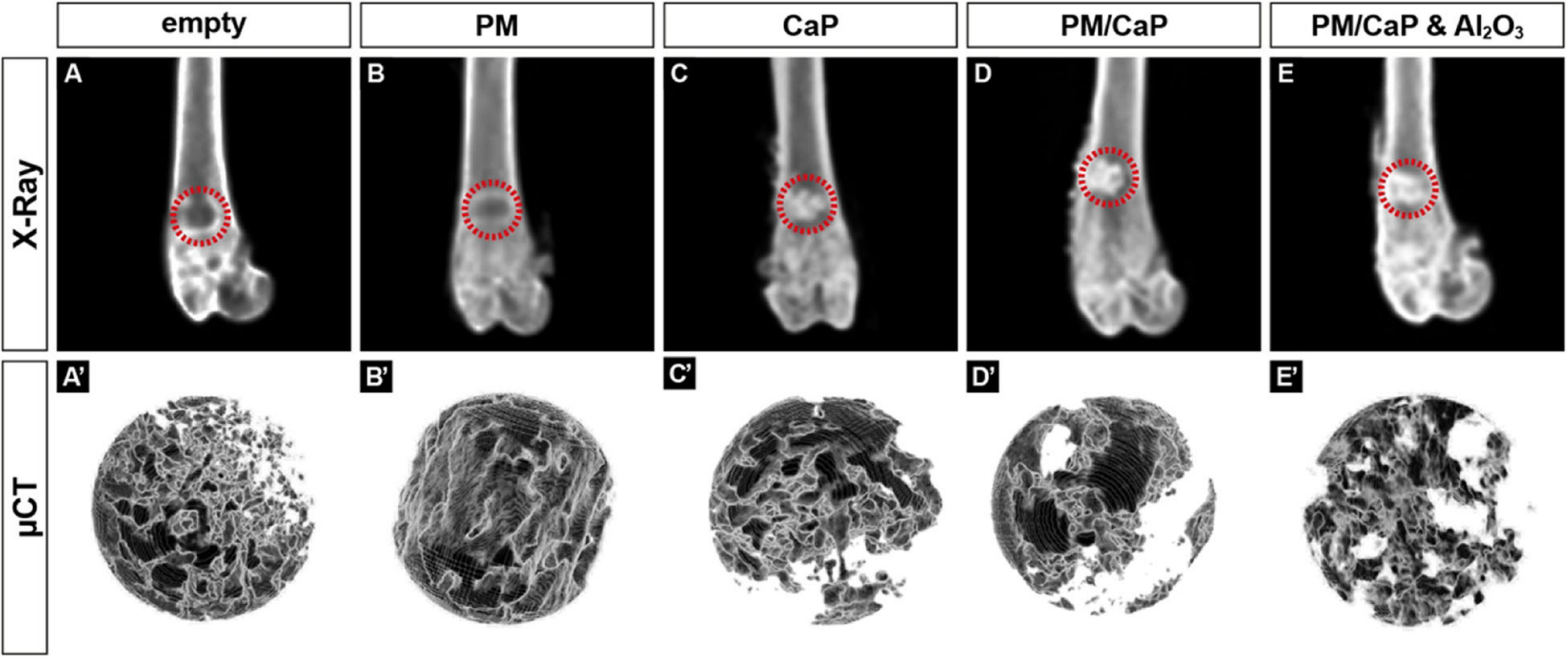
Representative X-ray and three-dimensional reconstructed quantified μCT data of the bone void treated with combination of materials. X-ray of the femora shows no ectopic bone formation

All drill hole defects were investigated by μCT, 4 weeks after operation (Fig. 5). Immediate X-ray following sacrifice confirmed that the drill hole defect in all bones was located in the metaphyseal part, while the opposite cortices were left untreated.

All groups which received the novel calcium phosphate-based material (CaP; PM/CaP and PM/CaP and Al_2_O_3_) showed an increase in relative bone volume (BV/TV) compared to the empty defect. This increase was significant between the groups that received CaP or CaP and Al_2_O_3_ compared to the empty defect group. Likewise, when comparing BV/TV percentage, there was a statistical significance across all groups compared to the PM group. The group that received PM/CaP and Al_2_O_3_ achieved the highest increase in BV/TV with a ratio of 88%.

**Fig. 6:**
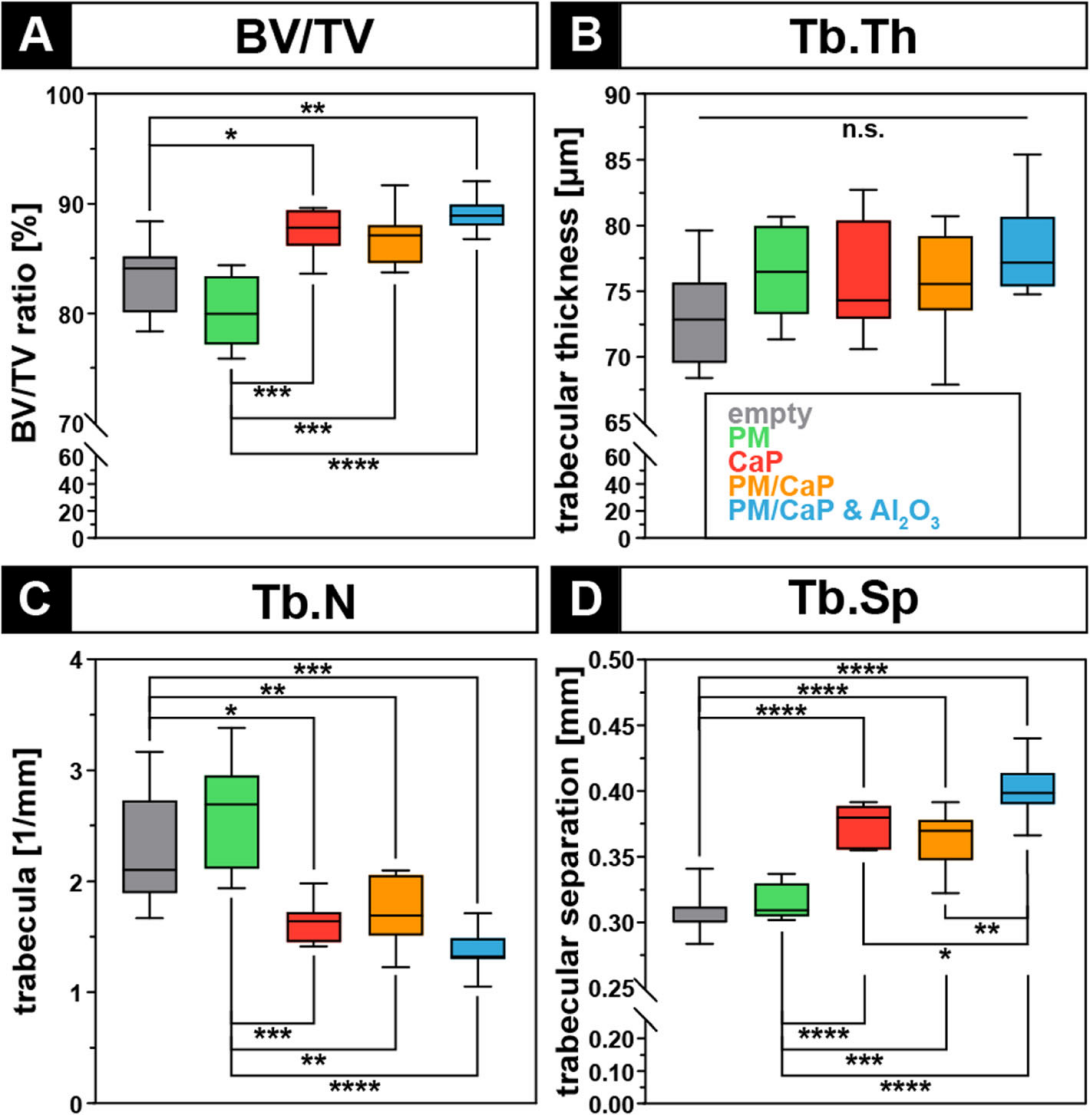
μCT analysis with a volume of interest located within the drill hole indicates higher osseous ingrowth in calcium phosphate-augmented defects. **a** Bone volume/total volume (BV/TV). **b** Trabecular thickness (Tb.Th). **c** Trabecular number (Tb.N). **d** Trabecular separation (Tb.Sp). *p ≤ 0.05; **p ≤ 0.01; ***p ≤ 0.001; ****p ≤ 0.0001. n.s., not significant

Further analysis into the trabecular microarchitecture revealed that the trabecular spacing (Tb.Sp) (Fig. 6d) showed also significant higher values in the groups which received CaP. Comparison of trabecular thickness (Tb.Th) (Fig. 6d) showed no significant changes. A homogeneous distribution was found across all groups.

The trabecular number (Tb.N) (Fig. 6c) in all CaP-treated groups was significantly decreased compared to empty or only PM-treated defects.

## Discussion

The present study was conducted to evaluate the biocompatibility and osteoconductivity of novel calcium phosphate-based composites that might shed to new strategies for the treatment of metaphyseal defects under in vivo conditions. Despite major research efforts, limited progress has been achieved in the field of new bone graft substitutes that offer the complex required needs, especially in older patients with osteoporotic fractures [18].

Clinically approved calcium phosphate-based biomaterials differ in their individual material properties and are selected to suit different clinical indications. These materials are in competition with PMMA cement, which has been proven safe and efficient in many applications for a long time. PMMA cement shows a high primary stability, but is under criticism regarding its biomechanical properties and the lack of biological transformation into bone tissue [19].

Substrates on beta tricalcium phosphate (beta-TCP) basis are in the meantime often used bone graft substitutes. They are commercially provided in porous blocks or wedges that can be introduced into the defect area. These clinically available materials have the disadvantage that accurate fitting into the defect can be challenging owing to their solid structure. Liquid or pasty materials that can be modeled to adapt the defect are therefore of increasing clinical interest. Also paste-like materials are already clinically available. Yet, larger clinical studies on universal applicability of these materials are still pending. Currently, one of these paste-like materials is primarily used for bone tumors, tibial head fractures, and osseous infections as a local antibiotic carrier [20]. These materials consist of two components and are mixed shortly before use. Essential components are hydroxyapatite and calcium sulfate. The consistency is suitable for a percutaneous injection. The calcium sulfate serves as a carrier for these materials, whereas in our experiments, a plant-based polysaccharide matrix was used. In addition, we added aluminum oxide, which is supposed to get hooked in the defect and thus, for example, withstand increased pressure, especially when used in vertebroplasty.

The animal model used in this experimental setup offers the advantage to verify biocompatibility and osteoconductivity [21]. In the present study, osseous ingrowth was observed by histology, as it was one of the major expectation. This observation was confirmed in μCT images in which the osseous ingrowth was more pronounced in CaP-treated defects compared to the plain defect or PM-treated specimens. Morphometric analysis via μCT supported these findings as all groups treated with CaP showed a significant increase in BV/TV. This is in accordance with other studies reporting a positive effect of CaP for bone regeneration [22, 23]. Koepp et al. also performed biomechanical tests in their in vivo study based on β-tricalciumphosphate (Cerasorb®). Here, the bones with the implanted materials showed greater strength compared to the controls. The authors attribute this to the higher volumetric density of β-TCP as compared to the control side and the strong connection between the host bone and the trabeculae transecting the implant. However, in samples treated with calcium phosphate, only a tendential increase in trabecular thickness was observed, associated with a reduced trabecular number and increased trabecular separation. We suspect that the biomaterial itself limits the interpretation of bony growth in the μCT, as an increase in Tb.N and a reduction in Tb.Sp would have been expected. Although μCT-based analysis was carried out in a standardized manner with consciously chosen scanning and rendering settings, it is likely that the biomaterial itself limits the interpretation of bony ingrowth within the drill hole defect during μCT analysis. Thus, the microarchitecture of the bone area and amount of composite material were accumulated, which limits the interpretation of bone ingrowth within the drill hole defect. In future studies, scanning with different settings such as dual energy absorptiometry could be beneficial, and also biomaterial enhancement with a contrast agent could provide improved interpretation of data in the future. The observed morphometric findings might also be attributed to the paste-like formulation of the matrix, which allows the precise application of the material and ensures that the material remains at the injection site. Schlickewei et al. also used an injectable form of bone replacement containing calcium phosphate and bisphosphonate, which offers advantages in handling [24]. As a new approach, alendronate was additionally added to the material combination to be tested. This was intended to improve implantation in the defect, but in this study, this had no advantage or disadvantage. The BV/TV was similar in both groups after 12 weeks, so that it can be assumed that the greatest effect is due to the CaP.

Thus, resorption of the CaP appears to gradually leave the space for osseous ingrowth, which in turn could have beneficial effects on stability. Further benefits of paste-like materials compared to pure CaP are particularly evident during application. Pure CaP granulates have to be stuffed into the drill hole defect, and the surgeon has limited technical possibilities to distribute the biomaterial in osseous defects of the surrounding tissue. Although changes of the osseous microstructure found in the present study in between the biomaterials are minimal, the application of the paste-like CaP offered fascilated injection during initial surgery. Thus, the biomaterial distributes easier into the surrounding osseus microstructure and provides osteoconductive properties not only in the central defect but also in the adjacent smaller bone voids.

A general disadvantage of bone graft substitutes at present is that no biomechanical stability, comparable to biological bone, is achieved [25]. In this context, the combination of HA and tricalcium phosphate used in the present study integrates two decisive advantages. HA is a natural component which is incorporated into regeneration as part of the natural bone metabolism [26]. In addition, tricalcium phosphate provides a fast biodegradation, compared to PMMA cement, but at the same time acts as a stabilizing component [27, 28]. Increased stability is necessary to reduce the failure rate following fracture fixation. Failure of osteosynthesis can be associated with revision surgery and a delay in mobilization, which triggers an increase in mortality [29]. In addition, older patients are not able to maintain weight-bearing restrictions, which requires immediate stability of the osteosynthesis [30]. In order to increase the biomechanical stability of the granular replacement matrix and to achieve the clinically required stability, Al_2_O_3_ was added in one group. Thus, aluminum oxide may have an extremely low degradation rate compared to CaP and thus, in case of a sufficient packing, may lead to a better stability than pure CaP. In previous in vitro test, Al2O3 demonstrated promising bone bonding properties while pairing with CaP appears to provide an addictive combination [31]. Naga et al. also observed good compatibility and new bone formation with an Al_2_O_3_-based scaffold. They also performed a biomechanical evaluation before implantation, but there was no biomechanical evaluation after the implantation [32]. Taken together, in the present study, we could show that a novel bone substitute consisting of a combination of CaP and Al_2_O_3_ has good in vivo and in vitro compatibility.

Limitations of this study were the small number of samples and the single time of analysis. During the in vitro experiments, further tests on the biocompatibility of the PM would have been desirable to serve as a control. Yet, due to the soft texture of PM, transfer of the cell/PM constructs was impeded and provided inconsistent data. Further studies are necessary to test these biomaterials in fracture fixation models, as well as in special models such as osteoporotic rats. A further limitation is the lack of investigation of the biomechanical properties of the materials and regenerates. Biotechnical tests are particularly important regarding the strength of the Al_2_O_3_-augmented granulates.

## Conclusion

In this study, we presented a new developed paste-like bone-filling biomaterial based on calcium phosphate granulates. We could show that our composite material can be easily applied by the surgeon under sterile conditions. Furthermore, we confirmed good biocompatibility under in vitro and in vivo conditions, such as an initiation of osseous ingrowth into the bone defect. The combination of a paste-like and solid calcium phosphate-based granulates could offer advantages in direct stability and consecutive osteoconductivity. Further research in large animal models of fracture healing as well as biomechanical testing is necessary to investigate the particular characteristics of this novel material.

## Data Availability

All data and materials were presented in the main paper and supplements attached.

## Abbreviations

BV: Bone volume
BW: Body weight
CaP: Calcium phosphate
HA: Hydroxyapatite
PM: Polysaccharide matrix
PMMA: Polymethylmethacrylate
ROI: Region of interest
SD: Standard deviation
Tb.N: Trabecular number
Tb.Sp: Trabecular separation
Tb.Th: Trabecular thickness
TCP: Tricalcium phosphate
TV: Tissue volume
VOI: Volume of interest
μCT: X-ray micro-computed tomography
