# Correction to: Bone defect reconstruction with a novel biomaterial containing calcium phosphate and aluminum oxide reinforcement

**DOI:** 10.1186/s13018-020-01945-7

**Published:** 2020-09-09

**Authors:** Alexander M. Keppler, Maximilian M. Saller, Paolo Alberton, Ines Westphal, Frank Heidenau, Veronika Schönitzer, Wolfgang Böcker, Christian Kammerlander, Matthias Schieker, Attila Aszodi, Carl Neuerburg

**Affiliations:** 1 Department of General, Trauma and Reconstructive Surgery, University Hospital of the Ludwig-Maximilians-University Munich, Campus Großhadern, Marchioninistraße 15, 81377 Munich, Germany; 2 Novartis Institute for Biomedical Research, Basel, Switzerland; 3 BioCer GmbH, Bayreuth, Germany; 4 LivImplant GmbH, Starnberg, Germany

**Correction to: J Orthop Surg Res (2020) 15:287**

**https://doi.org/10.1186/s13018-020-01801-8**

Following publication of the original article [1], the authors informed us that the given name and family name for the authors 3, 4, 5, 6, 7, 8, 9, 10 and 11 were switched.

The incorrect author name is: Alberton Paolo

The correct author name is: Paolo Alberton

The incorrect author name is: Westphal Ines

The correct author name is: Ines Westphal

The incorrect author name is: Heidenau Frank

The correct author name is: Frank Heidenau

The incorrect author name is: Schönitzer Veronika

The correct author name is: Veronika Schönitzer

The incorrect author name is: Böcker Wolfgang

The correct author name is: Wolfgang Böcker

The incorrect author name is: Kammerlander Christian

The correct author name is: Christian Kammerlander

The incorrect author name is: Schieker Matthias

The correct author name is: Matthias Schieker

The incorrect author name is: Aszodi Attila

The correct author name is: Attila Aszodi

The incorrect author name is: Neuerburg Carl

The correct author name is: Carl Neuerburg

The author group has been updated above and the original article [1] has been corrected.

## References

[1] Keppler AM (2020). Bone defect reconstruction with a novel biomaterial containing calcium phosphate and aluminum oxide reinforcement. J Orthop Surg Res.

